# On the self-management ability of peritoneal dialysis patients: a cross-sectional study with a mixed approach

**DOI:** 10.1371/journal.pone.0345323

**Published:** 2026-03-27

**Authors:** Juan Sun, Yifang Zhang, Miaomiao Zhao, Yongmei Wang, Yali Li, Huimin Zhang

**Affiliations:** 1 School of Nursing, Henan Medical University, Xinxiang, Henan, China; 2 Nursing Department, Henan Provincial Chest Hospital, Zhengzhou, China,; 3 Nursing Department, Henan Province Hospital of Tcm, Zhengzhou, China; Istanbul University-Cerrahpasa, Cerrahpasa Medical Faculty, TÜRKIYE

## Abstract

**Objective:**

To understand the current status and influencing factors of self-management ability in peritoneal dialysis (PD) patients.

**Methods:**

This study employed an interpretive sequential mixed-methods approach and followed the STROBE and CONDITION guidelines. From June 2024 to February 2025, convenience sampling was used to survey 507 PD patients from three tertiary hospitals. Quantitative data were collected using five scales. Concurrently, purpose sampling was used to conduct semi-structured interviews with 13 patients until data saturation was reached. Quantitative data were analyzed using SPSS 26.0, and qualitative data were analyzed thematically using Nvivo15. The results were integrated to provide a comprehensive understanding.

**Results:**

The quantitative results showed that PD patients had a self-management ability score of [55.00 (45.00, 60.00)] out of a possible 96, indicating a moderately low level. Multiple linear regression identified age, educational attainment, monthly household income, dialysis age, understanding of the disease, mastery of health education content, frequency of follow-up, and self-efficacy as significant influencing factors. The qualitative interviews identified two themes (obstructive and promoting factors) and nine sub-themes. The integration of results showed that qualitative findings confirmed and complemented the quantitative associations.

**Conclusion:**

The self-management ability of PD patients is moderately low. Medical staff should enhance the self-efficacy and social support of PD patients based on the influencing factors of their self-management ability, increase the frequency of follow-up, pay attention to PD patients with low education, advanced age and other types, strengthen training and monitoring for complications, and carry out targeted intervention to improve the self-management ability of PD patients.

## 1. Introduction

With the rapid aging of the population and changes in lifestyle, the incidence of chronic kidney disease (CKD) is also increasing year by year [1]. Due to the insidious onset of CKD and low awareness of the disease, patients are prone to various delays in seeking medical treatment, resulting in irreversible renal failure and eventual development of end stage renal disease (ESRD) [2]. Peritoneal Dialysis (PD) is an important treatment option commonly used for ESRD patients. It has the advantages of maintaining stable hemodynamics, helping to protect the residual renal function of patients, reducing the risk of cross-infection, effectively removing medium molecular toxins, simple equipment and convenient operation, and home-based treatment [3,4]. It has little impact on patients’ work and life, and is thus recommended as the preferred method of renal replacement therapy in many countries [5]. PD mainly takes advantage of the semi-permeable membrane properties of the peritoneum itself. Based on diffusion and convection mechanisms, dialysis fluid is injected into the abdominal cavity and then expelled to ultrafiltration excess water, remove metabolic waste accumulated in the body, and correct electrolyte and acid-base balance disorders [6].

Self-management is an important ability for patients to deal with chronic diseases, mainly including knowledge and skills of the disease, as well as emotional adjustment. The long-term efficacy of PD is not only dependent on the successful implementation of the initial catheter implantation technique, but also closely related to the standardized management of patients after discharge. The contents of self-management for PD patients include [7]: ① preventing infection, patients should pay attention to aseptic operation in self-management; ②PD catheter management, patients need to manage PD catheters effectively; ③ Volume management, strict control of water and salt intake; ④ Nutrition management, PD patients are prone to malnutrition, so nutrition management needs to be strengthened; ⑤Self-adjustment of psychological factors. Effective self-management reduces the risk of complications, guarantees treatment outcomes, and improves the quality of life and survival rate of patients [8]. Self-management education can also help PD patients have a correct understanding of the disease and enhance psychological adaptation and social reintegration, which is a key support for long-term survival [9].

Research shows that people with PD perform worse than those with common disease in terms of self-management ability [10]. Clinical research evidence shows that individual self-management ability is significantly associated with disease coping strategies, treatment compliance, and pathological outcomes [11,12]. Some studies have shown that 53.6% of PD patients have abnormal nutritional status, which is significantly associated with their self-management ability, leading to different electrolyte metabolic disorders that directly affect solute clearance efficiency and thereby reduce dialysis outcomes [13]. Therefore, this study will investigate the current status of self-management ability in PD patients and explore its influencing factors to provide a reference for future clinical intervention.

Quantitative studies have certain limitations, and it is not comprehensive enough to explore the factors that PD affects patients’ self-management ability. Therefore, in order to dig deeper into the factors influencing self-management ability in different categories of PD patients, this study adopted a mixed research approach, adding a qualitative research component on the basis of quantitative research. Through one-on-one in-depth interviews with PD patients, it complemented the quantitative research, increased the comprehensiveness and specificity of the study, and provided a reference for medical staff to formulate effective intervention measures.

## 2. Objective

This study aims to investigate the current status and influencing factors of self-management in peritoneal dialysis patients using a mixed-methods approach, providing a basis for medical staff to carry out high-quality self-management interventions. The specific aims are: ① to quantitatively explore the status and influencing factors of self-management in PD patients; and ② to qualitatively understand the self-management experiences and perceived factors from the patients’ perspectives.

## 3. Methods

### 3.1. General research design

This study employs an interpretive sequential mixed-methods approach, including both quantitative and qualitative research. The study design and reporting followed the Epidemiological Observational Study Reporting (STROBE) Declaration 30 (S1) and the Uniform Standard for Reporting Qualitative Studies (COSTO) guidelines (S2).

### 3.2. study settings

The study was conducted in three tertiary hospitals in Xinxiang City, Henan Province, China.

### 3.3. Sampling

#### 3.3.1. Quantitative section.

Using the convenience sampling method, patients treated in the nephrology departments of several tertiary grade A general hospitals in Xinxiang City, Henan Province were selected from June 2024 to February 2025, and questionnaire surveys were conducted among the research subjects according to the pre-established screening criteria. Inclusion criteria: ① Patients who had received Continuous Ambulatory Peritoneal Dialysis (CAPD) treatment for more than 3 months and whose condition was stable; ② Patients aged 18 years or older; [3] Voluntary participation in this study. Exclusion criteria: ① Accompanied by severe heart, liver, respiratory failure and malignant tumors; ② Severe mental confusion and inability to cooperate due to mental illness; ③ Participating in other research projects.

According to the rough estimation method, the sample size should be at least 5–10 times the number of variables to be measured [14]. There were 19 statistical variables in this study. Considering the possibility of invalid questionnaires or missing data during the implementation of the study, the sample base was expanded by an additional 20%. After comprehensive calculation, the final sample size required was no less than 238. A total of 560 questionnaires were distributed, and 507 were included in the final analysis.

#### 3.3.2. The qualitative part.

A purposive sampling method was used to ensure maximum variation in participant characteristics, including age, gender, educational level, and dialysis vintage. The inclusion criteria were consistent with the quantitative part. The sample size was determined by data saturation, the point at which no new themes emerged from the interviews. This was achieved with a final sample of 13 participants.

### 3.4 Data Collection

#### 3.4.1 Ethical Considerations and Consent Process.

The study follows the principles of the Declaration of Helsinki. The protocol was reviewed and approved by the Ethics Committee of Xinxiang Medical College (XYLL-20230292). For the quantitative part, participants were informed of the study's purpose, and written informed consent was obtained before they filled out the questionnaire. For the qualitative part, at the beginning of the interview, participants were again informed about the study and the audio recording. Verbal consent was obtained and audio-recorded prior to the start of the interview. All participants were informed of their right to refuse to participate or withdraw at any time without any negative consequences.

#### 3.4.2. Quantitative section.

A pre-survey was conducted in the ward before the questionnaire survey, including one self-designed questionnaire and four mature questionnaires. The self-designed questionnaire was developed by the researchers based on literature review and team discussion, and the tool was composed of two dimensions: demographic and sociological characteristics and disease-related data. The demographic characteristics included age, gender, educational level, etc. The disease-related data included primary disease, comorbidities, dialysis age, etc., totaling 16 items. The self-management ability of PD patients was evaluated using the peritoneal dialysis patient self-management scale developed by Pang Jianhong [15]. The severity of depressive symptoms in PD patients was quantified using the international psychological measurement scale patient health questionnaire (PHQ-9) [16]. Generalized anxiety disorder (GAD-7) was used to assess the clinical manifestations of anxiety symptoms in PD patients [17]. In this study, the chronic disease management self-efficacy scale developed by Lorig et al. was used to quantitatively assess the self-efficacy levels of PD patients [18]. The following scales were used, whose characteristics are summarized in Table 1 below.

**Table 1:** Description of Measurement Scales Used in the Study.

Scale Name (Author)	Construct Measured	No. of Items	Scoring & Interpretation	Validity & Reliability	Language
Peritoneal Dialysis Patient Self-Management Scale (Pang Jianhong, 2014)	Self-management ability	24	5-point Likert scale; Total score range 0–96. Higher scores indicate better self-management. Good/Poor categorization based on cut-offs [19].	Good content validity (CVI = 0.92); High internal consistency (Cronbach’s α = 0.91) [15,19].	Chinese
Patient Health Questionnaire-9 (PHQ-9)	Depression symptoms	9	4-point Likert scale; Total score range 0–27. Higher scores indicate more severe depression.	Well-validated; Cronbach’s α = 0.86 in this study.	Chinese
Generalized Anxiety Disorder-7 (GAD-7)	Anxiety symptoms	7	4-point Likert scale; Total score range 0–21. Higher scores indicate more severe anxiety.	Well-validated; Cronbach’s α = 0.89 in this study.	Chinese
Chronic Disease Self-Efficacy Scale (Lorig et al., 2001)	Self-efficacy	6	10-point Likert scale; Total score range 1–10. Higher scores indicate greater self-efficacy.	Good construct validity; High internal consistency (Cronbach’s α = 0.91) [18].	Chinese

Data collection was carried out by systematically trained professionals, and all investigators were required to complete the standardized training process, standardize the guidance language, focus on the principles of questionnaire content interpretation, and ensure accurate understanding of the key points of assessment in each dimension of the scale. A pre-experiment study was conducted before the formal implementation, and the structure of the questionnaire was optimized and the presentation of the items was adjusted based on the feedback from the pre-experiment. When collecting data on-site, the immediate quality control strategy was adopted. The investigators conducted completeness checks on the completed questionnaires, and on-site re-checks and supplementary entries were made for situations with logical contradictions or missing items. The data were entered and checked by two people.

#### 3.4.3. Qualitative section.

The interview outline was initially developed based on the different dimensions of the self-management ability scale. Three patients were pre-interviewed before the formal interview, and the interview outline was revised and improved. The researchers conducted one-on-one semi-structured interviews with the respondents at times when they had sufficient time. The interview was conducted in a demonstration room of the department, creating a quiet and pleasant environment for the interview. During the preparation stage of the interview, the researcher will give the interviewee a detailed account of the study's objectives, significance, and specific interview content, inform them that the research process will be fully recorded, and explain that the research will strictly adhere to the privacy protection mechanism, anonymize all collected data, and officially start the interview after thorough communication and obtaining the interviewee's consent. During the interview, the researcher maintained an objective and neutral stance, avoiding inducement or suggestion to the interviewee. To allow the respondents to fully express their true thoughts, the researchers employed interview techniques such as rhetorical questions, repetitions, and responses. The researchers used mobile phones to record and write down information such as changes in expression and body language. When no new information emerged during the interview, the data was considered saturated and the data collection session was terminated. The research team simultaneously transcribed the voice within 24 hours after each interview and organized and analyzed it in combination with the written records during the interviews. A summary of the interviews can be found in S3.

### 3.5. Data analysis and integration

#### 3.5.1. Quantitative section.

The questionnaire was entered using epidata3.1, and the data were statistically analyzed using IBM SPSS 26.0. Measurement data with normal distribution were expressed as mean ± standard deviation ($\overset{―}{x}$±s), while those with non-normal distribution were expressed as median (*M*) and quartile (*P*_*25*_, *P*_*75*_); Count data were represented by the number of cases and composition ratios (*n*, %). Patients’ self-management levels were classified based on their scores as follows: a total score of more than 67 was rated as “good”, 50–67 as “moderate”, and a total score of less than 50 as “poor” [19].

Since the self-management data were not normally distributed, non-parametric tests were used for comparison between groups; Spearman correlation analysis was used for the relationship between self-management ability and anxiety, depression, and self-efficacy. P < 0.05 was considered statistically significant.

#### 3.5.2. Qualitative part.

Within 24 hours of the end of the interview, transcribe the mobile phone recording data into a text version, confirm the questionable parts in the transcriptions to the interviewees, and form the final interview text data. Import the data into Nvivo15 software for data management and analysis. Data analysis: Read the transcribed text data repeatedly to familiarize yourself with the interview content; Look for words and phrases related to online learning ability to form the initial code; Form the initial code into different topics and check them, then merge, split, and delete some of the topics to meet the criteria of internal homogeneity and external heterogeneity; Review each topic again after determining it to reduce bias, and verify and confirm with the respondents again if in doubt.

#### 3.5.3. Integration.

After independent analysis of the quantitative and qualitative data, a parallel comparison approach was adopted to combine the results of the quantitative and qualitative parts. There are three types of relationships between quantitative and qualitative data: convergence, divergence and expansion [20].

### 3.6. Ethical considerations

The study follows the principles of the Declaration of Helsinki. The protocol of this study was reviewed and approved by the Ethics Committee of Xinxiang Medical College (XYLL-20230292). Participants were informed of the purpose of the study and signed the informed consent form. They have the right to refuse to participate in or withdraw from the study at any time without any negative consequences.

## 4. Results

### 4.1. Quantitative part

#### 4.1.1. Characteristics of the study sample.

A total of 560 questionnaires were distributed in this study, and 507 were effectively retrieved, with an effective recovery rate of 90.54%. The 507 PD patients in this study were aged 18–88[54 (45, 60)] years, with the majority being 40–60 years old, accounting for 51.28%. The gender ratio was average; The majority had primary school education and below, accounting for 63.51%; Other details are shown in Table 2. The depression score was [5.00 (2.00, 10.00)], the anxiety score was [3.00 (1.00, 7.00)], and the mental state was good. The self-efficacy score was [6.50 (5.50, 7.33)], which was relatively low.

**Table 2:** Scores of each dimension of self-management (*n* = 507).

Dimensions	Value range	Minimum value	Maximum	*M* (*P*_*25*_, *P*_*75*_)
Fluid change technique operation	0 ~ 21	2	21	16.00 (14.00, 19.00)
Handling of abnormal situations during operation	0 ~ 12	0	12	8.00 (6.00, 10.00)
Diet management	0 ~ 15	0	15	11.00 (9.00, 13.00)
Complication monitoring	0 ~ 24	0	24	11.00 (8.00, 15.00)
Emotion management and social return	0 ~ 12	0	12	6.00 (5.00, 8.00)

#### 4.1.2. Self-management ability of PD patients.

The self-management ability score of PD patients was [55.00 (45.00, 60.00)], which was at a moderately low level. The scores of each dimension are shown in Table 2. The top three items with the highest average scores were “correct connection and drainage of filtrate”, “correct venting and flushing of tubing”, and “use peritoneal dialysis fluid as prescribed”; The lowest three were “pay attention to limb weakness and numbness in hands, feet, and around the mouth,” “pay attention to chest pain, chest tightness, shortness of breath, palpitations, and increased weakness,” and “monitor for any unusual protrusions in the abdomen or groin.”

#### 4.1.3. Factors influencing self-management in PD patients.

Univariate analysis results showed that The 11 factors of age, educational level, place of residence, monthly household income, residence status, employment status, dialysis age, understanding of the disease, frequency of health education by medical staff, mastery of health education content and follow-up frequency had statistically significant effects on the self-management ability of PD patients (*P* < 0.05) (see Table 3).Self-management scores of PD patients were not correlated with depression and anxiety (*r* = −0.030, 0.054; *P* = 0.505, 0.229), but positively correlated with self-efficacy (*r* = 0.175, *P* < 0.001).

**Table 3:** Univariate analysis of factors influencing self-management ability in PD patients.

Variables	Number of cases [*n* (%)]	Total score of self-management ability *M* (*P*_*25*_, *P*_*75*_)/($\overset{―}{x}$±s)	*H*/*Z*	*P*
Age (years)			21.505^a^	<0.001
18 ~ 39	92 (18.15)	58.50 (53.00, 63.00)		
40 ~ 59	260 (51.28)	54.00 (45.00, 59.00)		
≥60	155 (30.57)	52.00 (43.00, 59.00)		
Gender			−0.429^b^	0.668
male	283 (55.82)	55.00 (45.00, 60.00)		
female	224 (44.18)	55.00 (46.50, 60.00)		
Educational attainment			47.044^a^	<0.001
Primary school and below	322 (63.51)	51.00 (42.00, 58.00)		
Junior high school or high school	87 (17.16)	58.00 (55.00, 61.50)		
College and above	98 (19.33)	57.00 (49.00, 64.00)		
Marital status			−1.261^b^	0.207
Married	433 (85.40)	54.00 (45.00, 59.00)		
Unmarried	74 (14.60)	56.00 (48.00, 61.00)		
Place of residence			10.208^a^	0.006
Rural	290 (57.20)	53.00 (44.00, 59.00)		
Town	141 (27.81)	56.00 (49.00, 61.00)		
Urban communities	76 (14.99)	55.00 (47.00, 60.00)		
Type of Health Insurance			2.908^a^	0.234
Urban residents	410 (80.87)	55.00 (46.00, 60.00)		
Workers	87 (17.16)	54.00 (42.50, 60.00)		
Out of pocket	10 (1.97)	39.00 (38.00, 56.00)		
Monthly family income (yuan)			27.888^a^	<0.001
1000 ~ 4999	158 (31.16)	51.50 (38.00, 58.00)		
5000 ~ 9999	236 (46.55)	55.00 (47.00, 59.00)		
≥10000	113 (22.29)	57.00 (52.00, 63.00)		
Status of Residence			11.789^a^	0.008
Living alone	38 (7.50)	55.50 (48.00, 60.00)		
Living with a spouse	325 (64.10)	55.00 (47.00, 59.00)		
Living with children	76 (14.99)	50.14 ± 13.37		
Others	68 (13.41)	60.00 (43.50, 64.50)		
Whether employed			−2.486^b^	0.013
yes	90 (17.75)	56.00 (49.00, 63.00)		
no	417 (82.25)	54.00 (45.00, 59.00)		
Dialysis age (months)			14.209^a^	0.001
3 ~ 11	79 (15.58)	49.00 (33.00, 58.00)		
12 ~ 23	83 (16.37)	54.00 (45.00, 58.00)		
≥24	345 (68.05)	55.00 (47.00, 61.00)		
Primary disease			6.562^a^	0.087
Glomerular diseases	180 (35.50)	53.00 (39.00, 60.00)		
Hypertension kidney damage	218 (43.00)	55.00 (47.00, 60.00)		
Diabetic nephropathy	82 (16.17)	55.00 (43.00, 59.00)		
Others	27 (5.33)	56.33 ± 10.65		
Comorbidities (individual)			7.605^a^	0.055
0	170 (33.53)	56.00 (50.00, 60.00)		
1	182 (35.90)	53.00 (43.00, 59.00)		
2	105 (20.71)	52.00 (45.00, 60.00)		
≥3	50 (9.86)	53.00 (41.00, 61.00)		
Degree of knowledge about the disease			13.442^a^	0.001
Not knowing	77 (15.19)	58.00 (52.00, 62.00)		
Relatively familiar	391 (77.12)	54.00 (45.00, 59.00)		
Know very well	39 (7.69)	59.00 (38.00, 63.00)		
The frequency with which healthcare workers carry out health education			36.259^a^	<0.001
＞1 once a month	128 (25.25)	51.50 (42.50, 62.00)		
Once a month	207 (40.83)	56.00 (50.00, 60.00)		
Once every 2 months	72 (14.20)	56.00 (53.00, 59.00)		
≥3 months/times	100 (19.72)	44.50 (32.00, 56.00)		
Mastery of health education content			10.800^a^	0.005
Less mastery	55 (10.85)	51.07 ± 13.95		
Half mastered	214 (42.21)	56.00 (50.00, 60.00)		
Totally mastered	238 (46.94)	52.00 (41.00, 60.00)		
Follow-up frequency			43.616^a^	<0.001
> once a month	125 (24.65)	52.00 (41.00, 62.00)		
Once a month	208 (41.03)	57.00 (50.00, 61.00)		
Once every 2 months	69 (13.61)	54.80 ± 4.64		
≥3 months/times	105 (20.71)	45.00 (34.00, 56.00)		

Note: a: *H*; b: *Z*

Multiple linear regression analysis (stepwise method) was conducted with the total score of self-management ability of PD patients as the dependent variable and the statistically significant factors in the univariate analysis as the independent variables. The variable assignment is shown in Table 4. The results showed that age, educational attainment, monthly household income, dialysis age, understanding of the disease, mastery of health education content, frequency of follow-up, and self-efficacy were correlated with the self-management ability score of PD patients (*P* < 0.05) (see Table 5).

**Table 4:** Assignment of variables.

Variables	Assignment
Age (years) Educational attainment	18 ~ 39 = 1; 40 ~ 59 = 2; ≥ 60 = 3 Primary school and below =1; Middle or high school = 2; College and above =3
Residence	Rural (*Z*_*1*_ = 1, *Z*_*2*_ = 0); Town (*Z*_*1*_ = 0, *Z*_*2*_ = 1); Urban communities (*Z*_*1*_ = 0, *Z*_*2*_ = 0)
Monthly household income (yuan)	1000 ~ 4999 = 1; 5000 ~ 9999 = 2; ≥ 10000 = 3
Residence status Whether employed Dialysis age (months) Knowledge of the disease How often healthcare workers carry out health education Mastery of health education content Follow-up frequency Self-efficacy	Living alone (*Z*_*1*_ = 1, *Z*_*2*_ = 0, *Z*_*3*_ = 0); Living with a spouse (*Z*_*1*_ = 0, *Z*_*2*_ = 1, *Z*_*3*_ = 0); Living with children (*Z*_*1*_ = 0, *Z*_*2*_ = 0, *Z*_*3*_ = 1); Other (*Z*_*1*_ = 0, *Z*_*2*_ = 0, *Z*_*3*_ = 0) Yes = 1, no = 2 3 ~ 11 = 1; 12 ~ 23 = 2; ＞ 24 = 3 Not knowing = 1; Better aware = 2; Very familiar = 3 > 1 times/month = 1; 1 time/month = 2; 2 months/time = 3; ≥ 3 months/time = 4 Less mastery = 1; Half mastery = 2; Full mastery = 3 > 1 times/month = 1; 1 time/month = 2; 2 months/time = 3; ≥ 3 months/time = 4 Actual values

**Table 5:** Multiple linear regression analysis of factors influencing self-management ability in PD patients.

Variables	Regression Coefficient *β*	Standard Error *SE*	Standardized regression coefficient *β′*	*t*	*P*	Tolerance	VIF
(Constants)	19.186	5.267		3.643	<0.001		
Monthly household income	2.802	0.691	0.166	4.053	<0.001	0.877	1.140
Educational attainment	4.518	0.629	0.294	7.186	<0.001	0.882	1.134
Dialysis age	2.884	0.666	0.177	4.332	<0.001	0.888	1.126
Self-efficacy divides evenly	1.577	0.375	0.180	4.202	<0.001	0.805	1.243
Follow-up frequency	−1.646	0.476	−0.142	−3.462	0.001	0.872	1.146
Age	−2.109	0.701	−0.118	−3.008	0.003	0.952	1.051
Mastery of health education content	2.913	0.853	0.159	3.417	0.001	0.679	1.474
Knowledge of the disease	3.310	1.143	0.128	2.896	0.004	0.757	1.321

Note: *R*_*2*_ = 26.5%, *ΔR*_*2*_ = 25.3%, *F* = 22.447, *P* < 0.001.

### 4.2 Qualitative part

#### 4.2.1 Characteristics of the respondents.

Semi-structured interviews were conducted with 13 PD patients, and the basic information of the respondents is presented in Table 6.

**Table 6:** General Information Status of Respondents (*n* = 13).

Number	Gender	Age (years)	Spouse	Educational attainment	Place of Residence	Average monthly income (yuan)
N1	Male	70	Married	3	Town	5000 ~ 9999
N2	Male	57	Married	2	Rural	5000 ~ 9999
N3	Male	56	Married	1	Rural	1000 ~ 4999
N4	Female	42	Married	2	Rural	5000 ~ 9999
N5	Male	30	Married	3	Urban communities	≥10000
N6	Female	54	Married	2	Rural	1000 ~ 4999
N7	Male	72	Married	1	Town	1000 ~ 4999
N8	Male	52	Married	2	Town	5000 ~ 9999
N9	Female	46	Married	3	Urban communities	5000 ~ 9999
N10	Male	79	Married	2	Urban communities	1000 ~ 4999
N11	Female	46	Married	3	Urban communities	5000 ~ 9999
N12	Male	64	Married	2	Rural	1000 ~ 4999
N13	Male	74	Married	2	Urban communities	1000 ~ 4999

Note: Primary school and below =1; Middle or high school = 2; College and above =3.

#### 4.2.2 Factors influencing self-management in PD patients.

Two themes, the hindrance factor and the facilitator factor, were extracted, and nine sub-themes were derived from the two themes.

#### 4.2.2.1 Hindrances.

(1) Improper operation

Strict aseptic operation is required during peritoneal dialysis, such as the preparation of the environment before the operation, the seven-step handwashing method and wearing a mask, but some patients tend to neglect these steps. For example, without a mask, bacteria in the mouth and nose cannot be isolated, which may lead to infection during peritoneal dialysis. **N1: “Sometimes I don't wear a mask properly.” “I think my own peritoneal dialysis is about the same as my own regular disinfection, wearing a mask or not, that's it.”**

(2) Lack of compliance

Some respondents showed a decline in compliance, which was manifested as failure to perform peritoneal dialysis and measure blood pressure on time. **N2: “Weigh yourself, sometimes and sometimes not. Weigh it every two days. Weighing every day is a bit annoying.” “The same goes for measuring blood pressure. Sometimes when it's cold, I don't measure it. It's not convenient to measure if you're not in good health.” N3: “It's too troublesome. It's a bit annoying to measure every day.” N4: “If the measurements are basically normal, I'll be lazy for a few days occasionally. I just say I'm not going out and I do it every day. I'm fine anyway. Unless I go back to my mom one day and then stop for one day, I don't stop as long as I don't go back.”**

(3) Bad mood

Peritoneal dialysis is a long-term treatment, and some patients are prone to negative emotions such as anxiety in the face of prolonged dialysis operations, which leads to negative management. **N3: “Sometimes a little irritable, this is the day after day.” “It's too troublesome, it's a bit annoying to test every day.” “Sometimes I get sick of it.”**

(4) Peritoneal dialysis time is tight

As peritoneal dialysis is performed multiple times a day and takes a long time, respondents say their jobs do not guarantee sufficient time for peritoneal dialysis, which reduces their self-management ability. **N5: “Going to work affects.” N8: “It's just that you have to work every day. After getting it done every day, you go to work in a panic, and then come back in a panic to do it again after working for the whole morning.”**

(5) Inadequate diet management

The respondents indicated that they lacked self-discipline and were unable to manage their diet as required due to family factors. **N5: “There might be a little bit of this kind of family meal.” N6: “Sometimes it does, because now the whole family eats together, and there are kids who are picky eaters. You cook, and if you like it, they may not.” N8: “I'm not very self-disciplined, and it's quite difficult for you to be perfect. Try to do as the doctor asks.”**

(6) Memory decline

Some respondents said they had poor self-management skills due to memory decline. **N7: “Forget, take medicine and measure blood pressure.” “For example, when it comes to this matter, you get up to change the fluid, you can measure the blood pressure while changing the fluid, and sometimes you can't remember to measure it. After changing the fluid, you forget to measure it again, and sometimes your memory is poor.”**

(7) Illness factors

The respondents said that the illness causes physical discomfort and affects their self-management. **N10: “I did as the doctor said, but I couldn't eat. I don't like to eat, I have anorexia, I don't want to eat anything I see.” “It's still the toxins that are too high and affect eating.” N12: “It hurts.” “It's just that the body doesn't get enough nutrition.”**

#### 4.2.2.2 Facilitators.

(8) Good social support

Respondents believe that social support is effective and can improve their self-management skills. **N5: “Family encouragement and encouragement between this friend is definitely effective.” N8: “The liquid changes are done in the courtyard where they live, and the environment is cleaner. My husband also urges me to pay special attention to this aspect.” N9: “I usually hear about peritoneal dialysis from my sons and daughters, and I haven't encountered any abnormal situations with peritoneal dialysis.” “Anyway, sometimes they tell me, my daughter and my son tell me. They all have that phone. Whatever happens at the hospital is sent to their phone, and he tells me at home.”**

(9) A positive attitude towards life

The core motivation of the respondents was family responsibility and survival needs that drove them to persist in treatment and self-management. **N11: “I haven't fulfilled this wish yet. Yes, and then another one, that kid is still young, isn't it? Anyway, there are plenty of things now.” “Nothing can stop it. Just live well.” N12: “For a better life, for a better family, you will do it on time.” “Look again at your role in life, in the family. There are parents and children who are not married, you are desperately following the doctor's instructions, I live a few more years to reduce the burden on the family. As an individual, I am insignificant. As a family, I strive for the people I live with, and I want to be strong.” N13: “The purpose of managing oneself is to survive.”**

### 4.3. Integration of quantitative and qualitative outcomes

There is consistency and complementarity between the quantitative and qualitative results. Qualitative research provides detailed evidence from the patients’ perspective, not only validating the statistical results of quantitative studies (such as the impact of age and education), but also deeply explaining how these factors specifically affect patient behavior (such as memory decline leading to operational errors). For details, please refer to Table 7.

**Table 7:** Integration of factors influencing self-management ability in different categories.

Factors	Quantitative results	Qualitative results	Quantitative and qualitative relationships
Obstructive factors	Old age, low educational attainment, low monthly household income	Improper operation, insufficient compliance, bad mood, tight peritoneal dialysis time, inadequate diet management, memory decline, disease factors	Some quantitative results are consistent with qualitative results, and qualitative results complement quantitative results
Facilitators	Longer dialysis time, more frequent follow-up, more knowledge of health education, better understanding of the disease, and higher self-efficacy	Good social support and a positive attitude towards life	The qualitative results complement the quantitative ones

## 5. Discussion

### 5.1. The self-management ability of PD patients is at a moderately low level and needs improvement

The score of self-management ability of 507 PD patients investigated in this study was [55.00 (45.00, 60.00)] points, which was lower than the research results of Li Huilin et al [21]., suggesting that their self-management ability needs to be improved. It may be related to the low educational attainment, insufficient understanding of the disease and inadequate mastery of health education among PD patients in this study. The top three items on the self-management ability scale for PD were all from fluid exchange techniques, while the bottom three were from complication detection, indicating that PD patients were more proficient in daily dialysis operations but had insufficient monitoring of complications. Home-based peritoneal dialysis is prone to various complications, while standardized complication management can improve self-management and dialysis quality [22]. Therefore, medical staff should attach importance to the management of complication monitoring in PD patients. They can conduct regular surveys such as remote monitoring to check whether PD patients measure their weight, blood pressure and intake and output daily, etc., to encourage patients to cooperate with treatment, reduce the occurrence of complications and improve their self-management ability [23]. Future longitudinal studies that incorporate objective measures of treatment efficacy, such as peritoneal Kt/V and residual renal function, would be valuable to explore the dynamic relationship between self-management ability and dialysis adequacy. Healthcare workers should also enhance communication with PD patients with low educational attainment and those living in rural areas, and focus on the effectiveness of health education for PD patients, carry out various forms of health education, and conduct assessment after health education for PD patients, thereby continuously improving their understanding of the disease.

### 5.2. Obstacles to self-management ability of PD patients

#### 5.2.1. Advanced age, low educational attainment, and low monthly family income.

The results of this study showed that older PD patients had poorer self-management skills, while younger patients had better self-management skills (*P* = 0.003), which is consistent with the results of related studies [24]. Younger patients had relatively stronger self-management abilities, possibly due to the better adaptability and acceptance of younger patients and the easier mastery of various operations. However, with age, degenerative changes in physiological functions (such as reduced motor coordination and weakened memory coding ability) and the cumulative effect of chronic diseases led to a gradient decline in self-management abilities, consistent with the results of qualitative partial memory decline. Therefore, it is recommended that healthcare workers, when intervening in older patients, repeatedly explain and demonstrate important information to enhance memory and improve the intervention effect, thereby enhancing their self-management ability. The lower the educational attainment of PD patients, the worse their self-management ability (*P* < 0.001) [25], the poorer their ability to learn and apply peritoneal dialysis knowledge, and the more difficult it is for them to master disease-related skills. It suggests that healthcare workers should focus on providing easy-to-understand education to patients with lower educational attainment to better help them manage the disease. PD patients with lower monthly family income have relatively lower self-management ability (*P* < 0.001), which may be due to the fact that PD patients have greater life pressure and often cannot work due to daily peritoneal dialysis, forcing their income sources to be cut off, further increasing life pressure [26]; On the other hand, low-income families have difficulty affording additional costs related to peritoneal dialysis, such as preparing a dedicated, safe and sterile peritoneal dialysis room, which increases the risk of infection and the likelihood of other complications, thereby affecting the self-management effect of patients. Therefore, medical staff should develop cost-effective treatment plans for low-income individuals, and relevant authorities can also consider providing assistance to low-income groups to ensure they have access to the necessary medical services and financial aid.

#### 5.2.2. Improper operation, insufficient compliance, and negative emotions.

Long-term follow-up data show that during home treatment, patients need to undergo dialysis fluid replacement 2–5 times a day. During this process, technical operation errors and poor care at the catheter outlet are the main risk factors for iatrogenic infections. Some studies have shown that improper dialysate replacement and bacterial infection at the outlet are the main causes of infection in PD patients [27]. Therefore, establishing standardized operation training programs based on evidence-based medicine is a key intervention to improve the self-management ability of home peritoneal dialysis patients. Due to the long treatment cycle of the disease and the individualized nature of the program, PD patients generally follow the standardized treatment process in the initial treatment stage. However, as the treatment cycle extends, the compliance level of some patients shows a downward trend, specifically manifested in non-standardized behaviors such as deviation in the execution of dialysis prescriptions and failure to follow the dietary requirements of medical staff [28]. To this end, medical staff can conduct regular phone follow-ups to investigate and urge PD patients to perform peritoneal dialysis and monitor complications, etc., in order to improve their self-management ability. Similar to the results of related studies, prolonged treatment can easily lead to negative emotions in patients, further affecting their compliance with medical advice and resulting in insufficient self-management ability [29,30]. Medical staff should pay attention to the mental state of PD patients, screen their mental state, and for those with negative emotions, provide them with multi-disciplinary collaborative services such as psychological counseling to improve their negative emotions.

#### 5.2.3. Peritoneal dialysis time is tight, diet management is insufficient, and disease factors.

Frequent peritoneal dialysis operations and long dialysis times may prevent working patients from ensuring adequate dialysis time. For this, PD patients can use automated peritoneal dialysis and try to use a circulation machine for dialysis at night, and work normally during the day. Or use a segmented peritoneal retention program, with scheduled fluid replacement periods, and perform fluid replacement during the morning, evening and lunch breaks. Scientific and reasonable diet management is crucial for maintenance peritoneal dialysis patients. Good dietary behavior helps to slow disease progression, reduce complications such as cardiovascular diseases, and improve long-term survival rate of patients [30]. Peritoneal dialysis requires long-term dietary management, which is a huge challenge for patients. Therefore, medical staff can look for suitable diet management software, recommend patients to use and record their own diet, and eat in advance in special circumstances such as family meals to improve self-control. Peritoneal dialysis patients have reduced or lost function of the residual nervous system, resulting in reduced excretion of water and toxins, followed by symptoms such as thirst, fatigue and loss of appetite, which increases the intensity of diet management and reduces their self-management ability [30]. The pain that occurs during the treatment has a dual negative effect: on the one hand, it intensifies the patient's resistance to the treatment; on the other hand, it activates the sympathetic – adrenal medullary system, causing abnormal secretion of adrenergic substances and triggering cardiovascular stress responses (including tachycardia and blood pressure fluctuations), affecting self-management [31]. Therefore, it is necessary to ensure that the patient receives adequate dialysis, actively treat the primary disease, and reduce the effects of toxins and pain.

### 5.3. Promoters of self-management ability in PD patients

#### 5.3.1. Longer dialysis duration and more frequent follow-up.

PD patients with longer dialysis duration had better self-management (*P* < 0.001) [32], which is consistent with the results of related studies. With the increase in dialysis age, patients accumulated more dialysis experience, gained a more comprehensive understanding of dialysis-related knowledge, improved operational norms for PD, were better able to accurately identify complications, and their self-management ability improved accordingly. In dialysis treatment, for patients in the early stage, medical staff need to strengthen health education and closely monitor the effect of the education. At the same time, the dialysis process should be tracked to help patients improve their self-management skills step by step. Patients who were followed up more frequently had better self-management (*P* = 0.001). In the long-term home peritoneal dialysis treatment of PD patients [33], the follow-up by medical staff can motivate patients to pay attention to their own health issues and provide health education during the follow-up, continuously improving the understanding of the disease among PD patients and thus enabling them to have high self-management ability. Therefore, medical staff should increase the frequency of follow-up visits and pay attention to examining the health education of PD patients. After providing health education to patients, they should ask them questions or conduct regular tests to urge PD patients to study the health education content and improve their self-management ability.

#### 5.3.2. Have a good grasp of health education content, have a good understanding of the disease, and have high self-efficacy.

The higher the level of mastery of health education, the stronger the self-management ability of the patients (*P* = 0.001), which is consistent with the results of related studies [34]. Therefore, continuing to enhance health education for patients is particularly important, especially for PD patients with low self-management ability. The better the understanding of the disease (*P* = 0.004), the clearer the understanding of the disease mechanism, treatment goals and operation norms, such as aseptic peritoneal dialysis patients being able to develop a scientific management plan to improve their self-management ability. For patients who have no knowledge of their own disease, medical staff should first identify the patients’ knowledge blind spots, and then carry out targeted and gradual health education to continuously improve their awareness of the disease.

Self-Efficacy is the subjective judgment of a person's ability to successfully carry out and achieve a behavioral goal and meet expectations, that is, confidence in one's own ability, which can influence an individual's behavior [35]. The higher the self-efficacy of PD patients, the better their self-management ability (*P* < 0.001). Wu et al. [36] conducted a cross-sectional survey of 247 patients in two nephrology clinics and dialysis centers, and the results showed that self-efficacy is an important factor influencing self-management behavior, and it plays a complete mediating role in the relationship between knowledge and self-management. In therapeutic care, in addition to providing patients with relevant knowledge, medical staff should also offer strategies to improve patients’ self-efficacy in order to enhance their self-care behavior and implement effective disease management.

#### 5.3.3. Good social support and a positive attitude towards life.

The results of this study show that social and family support helps patients manage themselves better, which is consistent with Xu Wenqi’s research [37]. Family care helps reduce patients’ negative emotions and encourages them to manage themselves. Therefore, when patients are discharged or come back for follow-up visits, medical staff should not only provide health guidance to patients but also encourage their families to support them and empower family supporters. Patients with a strong sense of survival tend to have a better mental state and be better able to manage themselves and have a stronger ability of self-management. It is suggested that medical staff should stimulate patients’ sense of survival to encourage them to manage themselves.

## 6. Conclusion

The self-management ability of PD patients is at a moderately low level, and the influencing factors involve multiple aspects such as demographic factors, disease-related factors, social factors, and psychological factors. Medical staff should formulate personalized intervention plans based on its influencing factors to enhance the self-management ability of PD patients with a particular focus on improving patients’ skills in monitoring for complications.. The deficiency of this study is that it is a cross-sectional study and the sources of PD patients investigated in this study are relatively concentrated. Furthermore, this study focused on CAPD patients; future research could include patients on automated peritoneal dialysis to compare self-management across different treatment modalities. In the future, a multi-center large-sample study can be carried out to comprehensively understand the self-management ability of PD patients, implement targeted measures, and lay the foundation for exploring long-term and effective strategies for cultivating and improving self-management ability.
